# Environmental factors controlling the distributions of *Botryococcus braunii* (A, B and L) biomarkers in a subtropical freshwater wetland

**DOI:** 10.1038/s41598-018-26900-9

**Published:** 2018-06-05

**Authors:** Ding He, Bernd R. T. Simoneit, Rudolf Jaffé

**Affiliations:** 1 Institute of Environment & Biogeochemistry (eBig), School of Earth Science, Zhejiang University, Hangzhou, 310027 China; 2 Marine Science Program and Southeast Environmental Research Center, Florida International University, Miami, FL 33199 USA; 3 Department of Chemistry, College of Science, Oregon State University, Corvallis, OR 97331 USA; 4 Department of Chemistry & Biochemistry, Florida International University, 3000 NE 151st St., North Miami, FL 33181 USA

## Abstract

Here we report the molecular biomarker co-occurrence of three different races of *Botryococcus braunii* (*B. braunii*) in the freshwater wetland ecosystem of the Florida Everglades, USA. Thespecific biomarkers include C_32_–C_34_ botryococcenes for race B, C_27_–C_32_ *n*-alkadienes and *n*-alkatrienes for race A, and lycopadiene for race L. The *n*-alkadienes and *n*-alkatrienes were present up to 3.1 and 69.5 µg/g dry weight (dw), while lycopadiene was detected in lower amounts up to 3.0 and 1.5 µg/g dw in periphyton and floc samples, respectively. Nutrient concentrations (P and N) did not significantly correlate with the abundances of these compounds. In contrast, *n*-alkadienes and *n*-alkatrienes were present in wider diversity and higher abundance in the floc from slough (deeper water and longer hydroperiod) than ridge (shallower water and shorter hydroperiod) locations. *n*-Alkadienes, *n*-alkatrienes, and lycopadiene, showed lower δ^13^C values from −40.0 to −35.5‰, suggesting that the source organisms *B. braunii* at least partially utilize recycled CO_2_ (^13^C depleted) produced from OM respiration rather than atmospheric CO_2_ (^13^C enriched) as the major carbon sources.

## Introduction

The green alga *B. braunii* is widely distributed in aquatic ecosystems, especially lakes and ponds^1^. The Botryococci are known to contain high amounts of a remarkably diverse range of unusual hydrocarbons, such as botryococcenes, *n*-alkadienes and *n*-alkatrienes, C_40_ monoaromatic compounds including lycopadienes and related oxygenated compounds that provide source diagnostic information^2–5^. While the C_23_–C_33_ *n*-alkadienes and *n*-alkatrienes, and squalenes (less specific) were reported as indicators of race A of *B. braunii*^5–7^, botryococcene (C_30_–C_37_) related lipids and methylated squalenes (C_31_–C_34_) were believed to be specific biomarkers biosynthesized by race B of *B. braunii*^2,4,5^. In contrast, race L contains isoprenoid structures related to lycopadiene^8–10^. These biomarker compounds, especially the saturated forms of botryococcenes and lycopadieneswell preserved in sediments and rocks, were thus used as biomarkers for paleoreconstructions^5,9^.

The Florida Everglades is the largest, subtropical freshwater wetland in the United States. Since the early 20^th^ century it has been drained significantly because of structural modifications for flood control, urban and agricultural development, which severely reduced its size, and over 5,000 km^2^ (50%) of the original landscape has been converted to agricultural and urban use during the last half century. Drainage of the wetlands resulted in shifts in the composition and distribution of vegetation cover, changes of the water quality and hydroperiod^11^. Currently, the vegetation shifts along the Everglades landscape from sloughs (deeper water, longer hydroperiod) with emergent and submerged plants, to ridges (shallow water, shorter hydroperiod) with *Cladium* sp. dominated communities, and scattered tree islands dispersed throughout this landscape^12^. Within this diverse distribution of plant species, periphyton mats, composed of abundant calcareous mixtures of diatoms, cyanobacteria and green algae, are distributed widely throughout this ecosystem^13,14^. In the Everglades, periphyton occurs primarily as benthic or floating mats instead of free floating phytoplankton. Thus, the suspended particulate organic matter is mostly found as flocculent material (floc), which consists of a non-consolidated layer of microorganisms, organic (detritus and disaggregated periphyton remains) and inorganic particles^15^.

Although *B. braunii* is distributed widely in aquatic ecosystems, especially in tropical oligotrophic freshwater to brackish lakes^16^, the microfossils of *Botryococcus* have only been reported in soil cores of tree islands in the Everglades^17^. A series of botryococcenes with carbon numbers from C_32_ to C_34_ were also detected in periphyton, floc, surface and deeper soils across the Everglades wetlands^18^, suggesting the existence of race B of *B. braunii*. Although individual races of *B. braunii* are widely distributed, reports of the co-existence of the different chemical races are rare. To our best knowledge, there is only one prior report of the co-existence of three races in a freshwater crater lake^1^. Here, we report the molecular characterization of various tracers of three races of *B. braunii* including botryococcenes, long chain *n*-alkadienes, *n*-alkatrienes, and lycopadiene in environmental samples of the Everglades ecosystem, examine their stable carbon isotopes, and discuss possible controlling factors including nutrients and hydroperiod on their distribution and abundances.

## Experimental Methods

### Sampling locations

**Figure 1:**
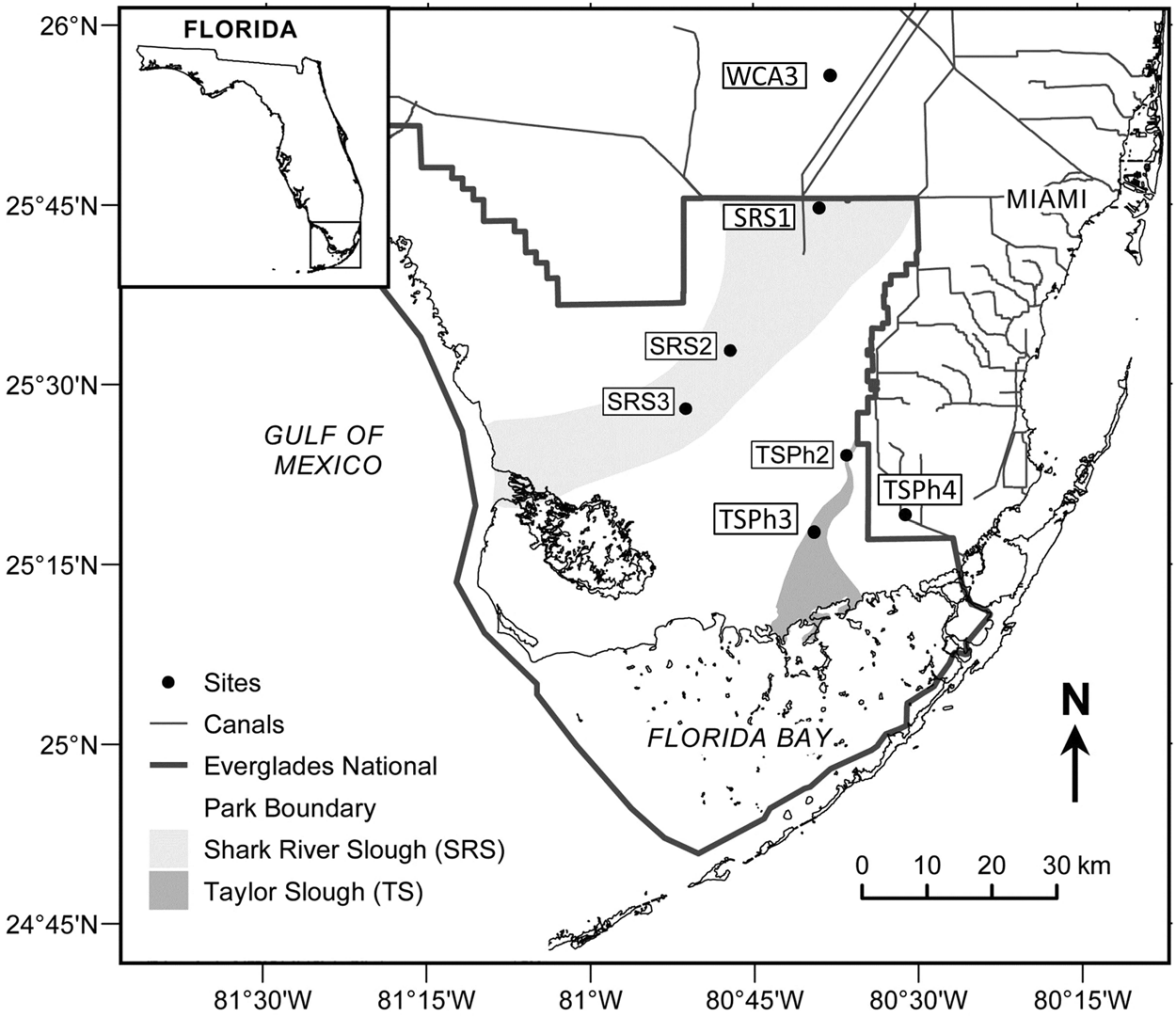
Map of the study area and sampling locations across the Florida Everglades wetland ecosystem. Sampling sites are marked with black dots. The map figure was generated by using software Google Earth (open-access version: 7.1.5.1557) (data was available from Data SIO, NOAA, U.S. NAVY, NGA, GEBCO, Image@2017 TerraMetrics) and then drafted by using software CorelDRAW (Graphics Suite × 6, source ID: 017002) (http://www.coreldraw.com/en/product/graphic-design-software). The sampling sites were located by using Global Positioning System (GPS).

The sampling sites for this study feature a gradient of nutrient and hydroperiod in the Everglades (Fig. 1; Table 1; http://fcelter.fiu.edu/data)^19^. Briefly, Water Conservation Area 3 (WCA3) is located to the north of Everglades National Park (ENP),and has the longest hydroperiod and highest nutrient (P and N) levels among all freshwater sites. Sites SRS1 to SRS3 are located in the Shark River Slough within ENP, characterized by intermediate hydroperiod and nutrient levels. Sites TSPh2–4 are located in the Taylor Slough within ENP, with lower hydroperiod and nutrient levels (Table 1). All these sites (SRS1–3 and TSPh2–4) are characterized by diverse aquatic vegetation and microbial communities^12^.

**Table 1:** Environmental data among different sites in this study.

Sampling locations	Hydroperiod (day)^a^	Surface water TN (µM)	Surface water TP (µM)	Floating Periphyton (%/m^2^)^c^	Benthic Periphyton (%/m^2^)^c^	Epiphytic Periphyton (%/m^2^) ^c^
WCA3	354	119.3^b^	0.45^b^	N.A.	N.A.	N.A.
SRS1	357	73.00^c^	0.35^c^	34.4	19.7	57.5
SRS2	327	69.50^c^	0.28^c^	31.2	7.0	33.6
SRS3	296	60.70^c^	0.37^c^	9.0	4.2	26.5
TSPh2	223	34.78^c^	0.20^c^	3.6	44.6	42.2
TSPh3	N.A.	52.04^c^	0.17^c^	7.6	56.4	56.5
TSPh4	N.A.	27.48^c^	0.20^c^	N.A.	N.A.	N.A.

Note: ^a^Data obtained from Saunders *et al*., 2015.

^b^Data from South Florida Water Management District.

^c^Data from FCE-LTER http://fcelter.fiu.edu/data.

N.A. = not available.

### Sample collection and preparation

Periphyton and floc (regardless of ridge and slough environments) were collected from various locations across the Florida Everglades (Table 2). Additional floc samples were sampled from both ridge and slough environments, and during different times of the year from 2012 to 2014 within WCA3. Both submerged and floating periphyton were placed into Ziploc bags. Floc, surface soils were sampled following the procedures as described previously^20,21^. Both leaves and roots (when applicable) of the dominant plants such as *Nymphaeaceae*, *Utricularia* sp., *Chara* sp., *Cladium* sp., *Eleocharis* sp., *Typha domingensis* P., and *Typha latifolia* L. were randomly sampled from different locations within ENP and WCA3^22^. All samples were kept cool on ice during transport to the laboratory. The samples were transferred into pre-combusted glass jars and stored at −20 °C until further analysis.

**Table 2:** Average concentrations of *n*-alkadienes, *n*-alkatrienes and lycopadiene detected in typical periphyton and floc across the Everglades freshwater wetlands (ng/g dw). Compounds listed according to sequential retention time.

Compounds	Kovats retention indexes^a^	Periphyton							Floc		
		WCA3 (n = 4)	SRS1	SRS2	SRS3	TSPh2	TSPh3	TSPh4	WCA3 (n = 86)	SRS2 (n = 6)	TSPh2 (n = 6)
C_27:3_	2630	—	—	—	—	—	—	—	347 (275)	—	45 (52)
C_27:2_	2643	—	—	—	—	—	—	—	103 (97)	—	—
C_28:3_	2727	—	—	—	—	—	—	—	658 (493)	—	—
C_28:2_	2744	—	—	—	—	—	—	—	168 (107)	—	—
C_29:3_	2826	7981 (2457)	—	—	—	—	—	—	9838 (2587)	918 (573)	—
C_29:2_	2843	1957 (974)	—	2887	169	129	694	—	1267 (871)	519 (369)	247 (189)
C_30:3_	2923, 2927	—	—	—	—	—	361	—	4560 (3517)	1562 (975)	537 (371)
C_30:2_	2941, 2965	—	1676	768	15	82	—	753	1505 (719)	584 (347)	129 (91)
C_31:3_	3022	—	—	—	—	—	—	—	1377 (439)	1198 (813)	307 (254)
C_31:2_	3040	4521 (721)	201	343	—	—	1312	66	3434 (1027)	—	—
C_32:2_	3070, 3110	—	129	—	—	—	—	—	2430 (1316)	—	—
Lycopadiene	—	1121 (894)	44	1845	168	974	2970	513	—	91 (78)	69 (51)

Note: all numbers are normalized by ng/g dw (sample); “−” = not determined. See Fig. 1 for locations and abbreviations of sample names; ^a^all retention indeces are calculated based on Rtx-1 column from Restek, USA.

All samples were processed and analyzed as described previously^21^. Briefly, they were freeze-dried at −50 °C, then shredded and sieved through a 32 mesh (500 µm) sieve to remove coarse material. Samples (1–3 g dry weight) were subjected to ultrasonic extraction three times (0.5 h each) with dichloromethane (DCM) (Optima, Fisher, USA) as solvent. Total extracts were concentrated and then fractionated by adsorption chromatography over silica gel. The aliphatic fraction and aromatic hydrocarbon fraction were eluted sequentially using *n*-hexane and hexane: toluene (3:1, v:v), respectively. Ziploc bags used for sampling were washed with *n*-hexane and the extracts were employed as control blanks and randomly analyzed to exclude external contamination.

### Bulk parameter analysis

Total nitrogen (TN) was measured by the high-temperature dry combustion method using a Carlo-Erba NA-1500 CNS Analyzer. Total P was analyzed with a Technicon Auto Analyzer II System (Pulse Instruments Ltd.), according to the standard method for orthophosphate P (EPA method 365.1). Bulk δ^13^C values were also determined for floc samples using standard elemental analyzer isotope ratio mass spectrometer (EA-IRMS) procedures^23^, and reported with respect to the Vienna PeeDee Belemnite (VPDB) standard for carbon. Precision of the δ^13^C measurements was ±0.10‰.

### Gas chromatography-mass spectrometry (GC-MS)

GC-MS analyses were performed on a Hewlett-Packard 6890 GC fitted with a Rtx-1 capillary column (30 m, 0.25 mm ID, Restek, USA) interfaced to a HP 5973 MSD. Compounds were quantified by squalane as the internal standard, assuming a similar response factor. Kovats retention indexes (RI)^24^ were calculated following the formula: RI = 100 × (R_*x*_−R_*n*_)/(R_*n+1*_−R_*n*_) + 100*n*, where x denotes the compound of interest, R denotes the GC retention time, and *n* and *n* + 1 denote the carbon number for the nearest *n*-alkane and (*n + *1)-alkane eluting before and after x, respectively on the GC. The identification of individual compounds was based on the comparison with published mass spectra and interpretation of the mass spectra^1^.

### Gas chromatography-isotope ratio mass spectrometry (GC-IRMS)

The δ^13^C values of individual *n*-alkadienes, *n*-alkatrienes and lycopadiene were measured using a GC-IRMS system, which consists of a HP 6890 GC equipped with a Rtx-1 fused silica capillary column (30 m, 0.25 mm ID), a combustion interface (Finnigan GC combustion IV), and a Finnigan MAT delta Plus mass spectrometer^21^. Between every three samples, three standard mixes containing squalane and C_17_ *n*-alkane (different concentrations as 30 ng/µL, 100 ng/µL and 500 ng/µL, with known δ^13^C values for each compound) were analyzed to check instrument performance and also for correction purposes. A known amount of squalane was used as an internal standard. The δ^13^C values are given in the per mil (‰) notation relative to the Vienna PeeDee Belemnite (VPDB) standard. The reproducibility of the GC-IRMS system was <0.5‰ for both standards and repeat analyses of selected samples (n = 3). Due to the co-elution of a few *n*-alkadiene or *n*-alkatriene isomers, and the relative lower concentration for some specific non-dominant isomers, only compounds present in sufficient quantities (intensity above 1000 mVs) could be accurately determined for reliable δ^13^C values. Average values were reported if more than one δ^13^C value was measured for isomers with the same carbon atom numbers.

### Data analysis

Environmental data across multiple locations was obtained from the Florida Coastal Everglades Long Term Ecological Research database (FCE-LTER; http://fcelter.fiu.edu/) and used for comparison with the abundance of the biomarker compounds (botryococcenes, *n*-alkadienes, *n*-alkatrienes, and lycopadiene). Statistical analyses were performed using SPSS version 13.0 for Windows. Outliers were tested using the two-sided Grubbs test (*P* < 0.05). Significant correlations (*P* < 0.05) between floc physicochemical parameters and the biomarker compounds were determined using Pearson correlation. Significant differences between means of different groups of data were compared using the unpaired t-test (two-tailed, unequal variance).

## Results

### Identification of *n*-alkadienes, *n*-alkatrienes and lycopadiene

**Figure 2:**
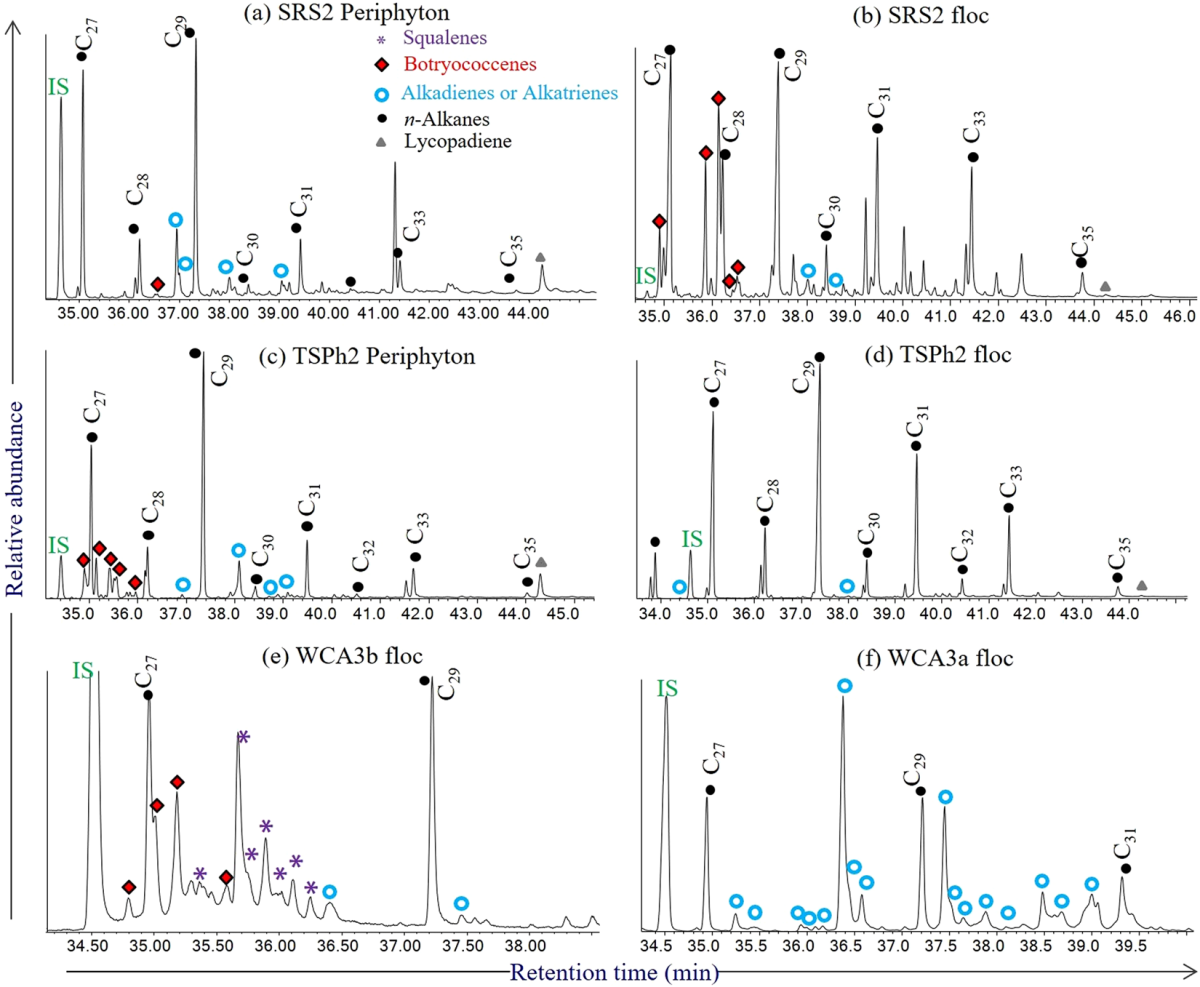
GC-MS data for a typical periphyton (**a**) and floc (**b**) sample from SRS2, a typical periphyton (**c**) and floc (**d**) sample from TS2, and typical floc samples from WCA3a (**e**) and WCA3b (**f**) (aliphatic fraction, partial TIC trace). The *n*-alkadi(tri)enes, botryococcenes, lycopadiene and *n*-alkanes are indicated by blue circles, red diamonds, gray triangles and black dots, respectively.

**Figure 3:**
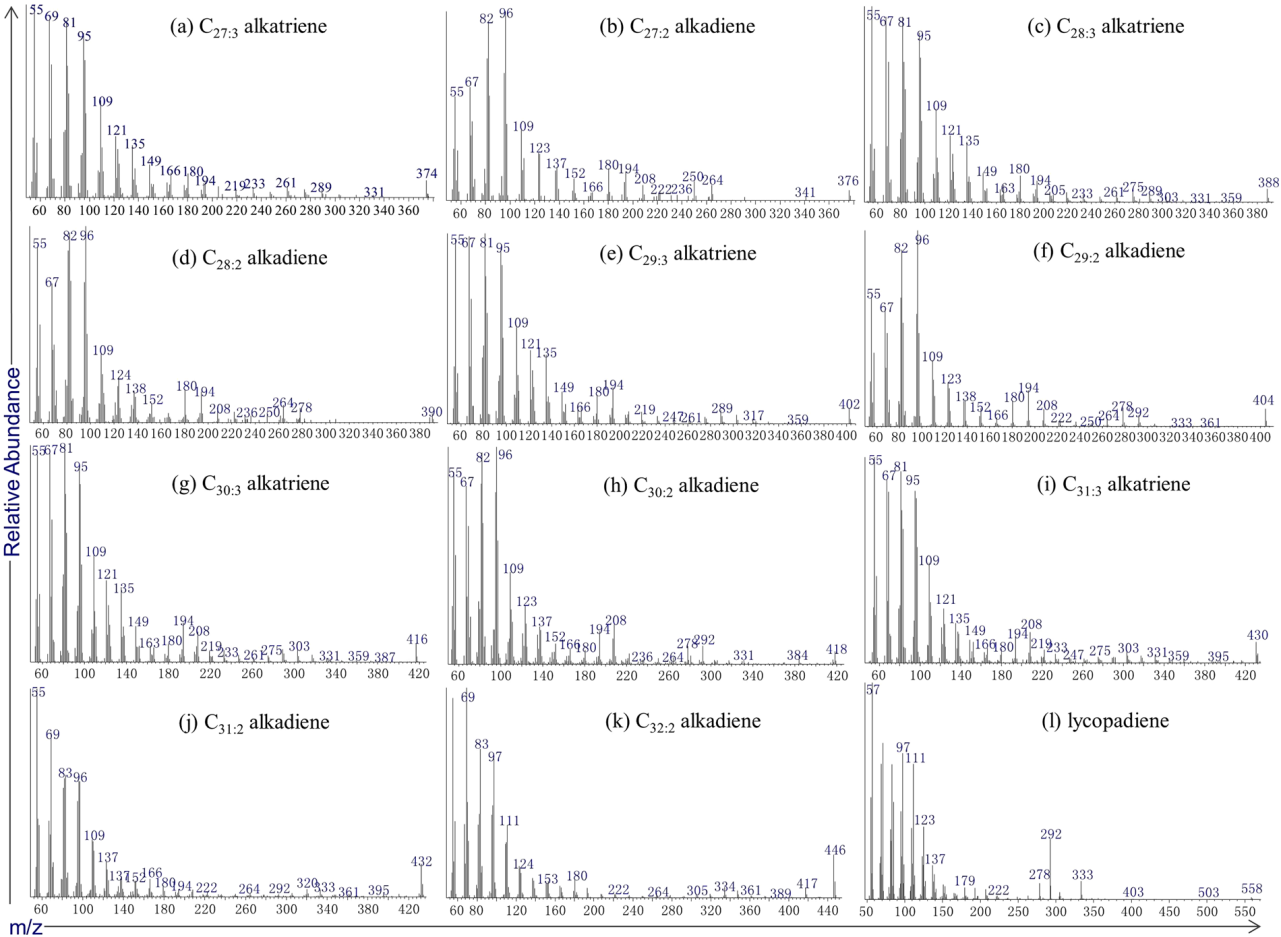
Examples of mass spectra of typical *n*-alkadienes, *n*-alkatrienes and lycopadiene identified in this study. Compounds given according to sequential retention time. All identifications are tentative.

GC-MS analysis of the *n*-hexane eluted fraction from various periphyton and floc sample extracts showed the presence of *n*-alkadienes, *n*-alkatrienes and one lycopadiene (Fig. 2; Table 2). A total of 11 C_27_ to C_32_ *n*-alkadiene and *n*-alkatriene isomers eluting between the C_26_ to C_32_ *n*-alkanes were tentatively identified and their Kovats indexes are also given (Figs 2a–f; 3a–k; Table 2). These compounds all exhibit a terminal double bond and one or two mid-chain unsaturations with both Z and E stereochemistry^1^. The mid-chain double bond positions could be further identified based on dimethyl disulfide adducts experiments^25^, but this is not pursed in this present study. Generally, no carbon number predominance was found for these *n*-alkadienes and *n*-alkatrienes. Similar no odd or even carbon chain predominance was observed in C_37_–C_43_ mono-, di- and (to a lesser extent) tri-unsaturated *n*-alkenes reported in lacustrine sediments^26^. Lycopa-14,18-diene was identified based on its retention time and mass spectrum match with that in the literature (Fig. 3)^1,9^. Another lycopadiene isomer with lower abundance was also identified (Fig. 3). A series of botryococcenes with 32 to 35 carbon atoms are detected in most periphyton and floc samples and elute between C_26_ to C_29_ *n*-alkanes, in agreement with a previous report (Fig. 2)^18^.

### Spatial distribution of *n*-alkadienes, *n*-alkatrienes and lycopadiene

**Figure 4:**
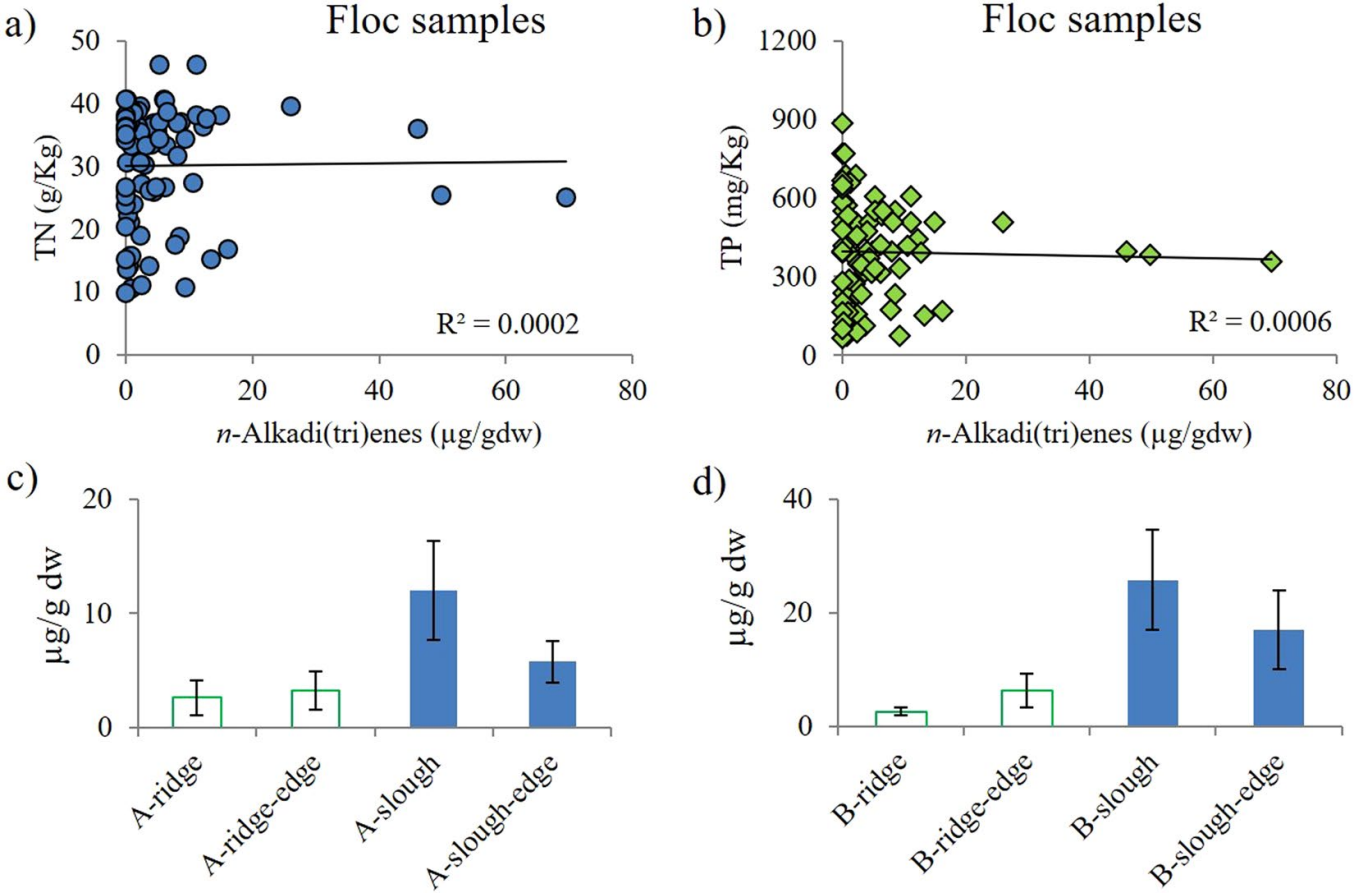
Multiple possible controlling factors for the distribution of *n*-alkadienes and *n*-alkatrienes in floc samples. (**a**) *n*-alkadi(tri)ene concentration data vs. Total N concentrations; (**b**) *n*-alkadi(tri)ene concentration data vs. Total P concentrations; (**c**) average concentrations of *n*-alkadi(tri)enes across ridge to slough transects for location A within area WCA3; (**d**) average concentrations of *n*-alkadi(tri)enes across ridge to slough transects for location B within area WCA3. Note: the average concentrations of *n*-alkadi(tri)enes for Fig. 4c,d were based on four sampling events during Oct. 2012, Jan. 2013, Oct. 2013, and Jan. 2014, respectively.

A higher molecular diversity of *n*-alkadienes and *n*-alkatrienes was detected in floc compared to periphyton (Table 2). Specifically, only C_29_ and C_31_ *n*-alkadienes, and C_30_ *n*-alkatriene were found in periphyton samples, while C_27_–C_31_ *n*-alkatrienes were present in floc samples. Lycopadiene occurred in most of the periphyton samples, but rarely in floc samples. Floc samples (n = 86) from both ridge and slough locales within the WCA3 area and floc samples (n = 12) from SRS2 and TSPh2 were analyzed. The N and P (nitrogen and phosphorus) concentration of these floc samples were 9.7–46.2 mg/g dw and 73–884 µg/g dw, respectively. The total concentration of *n*-alkadienes and *n*-alkatrienes of these floc samples ranged from 135 to 6953 ng/g dw. Surprisingly, the abundance of the C_29_ *n*-alkatriene could be up to 2 times above that of the C_29_ *n*-alkane in the same sample (Fig. 2), which is in contrast with previous reports that *n*-alkadienes and *n*-alkatrienes usually show much lower abundances than the odd numbered n-alkane homologues^1^. No significant correlations were observed (*P* > 0.05) between nutrient concentrations and the concentrations of each compound group or the total concentrations in floc (Fig. 4). In addition, no significant correlations were observed between surface water nitrogen and phosphorus concentrations, and abundances of *n*-alkadienes and *n*-alkatrienes among different locations across the freshwater wetland.

Floc samples from ridge (n = 19) and slough (n = 12) environments (within 5 m distance) were analyzed from multiple years (2012 to 2014) within the WCA3 area. The concentrations of *n*-alkadienes and *n*-alkatrienes in the slough floc samples ranged from 2.0 to 69.5 µg/g dw (average as 13.9 µg/g dw). In contrast, the concentrations of *n*-alkadienes and *n*-alkatrienes in the ridge floc samples ranged from 0 to 6.6 µg/g dw (average as 1.6 µg/g dw; Fig. 4). The concentrations of *n*-alkadienes and *n*-alkatrienes were significantly higher in the slough than the ridge floc (unpaired student t-test, two tailed, *P* < 0.01). In addition, 8 transects were analyzed from slough to the ridge environments (n = 32) and obvious concentration decrease trends were observed (Fig. 4).

### Compound specific carbon isotopes of *n*-alkadienes, *n*-alkatrienes and lycopadiene

Compound specific stable carbon isotope analysis was performed on the dominant *n*-alkadienes, *n*-alkatrienes and lycopadiene. Due to incomplete GC resolution of some *n*-alkadiene or *n*-alkatriene isomers with the same carbon number, the δ^13^C values are reported as averages for those compounds (mixtures of Z and E isomers). Significantly lower δ^13^C values were observed for the *n*-alkadienes, *n*-alkatrienes and lycopadiene (Table 2) than those of *n*-alkanes (−32.7 ± 1.8‰) and bulk samples (−30.7 ± 1.4‰). No significant differences in the averaged δ^13^C values were observed between *n*-alkadienes and *n*-alkatrienes, whereas the averaged δ^13^C values of the *n*-alkadienes and *n*-alkatrienes were lower than those of lycopadiene (Table 3).

**Table 3:** Compound specific carbon isotope compositions of selected *n*-alkadienes, *n*-alkatrienes and lycopadiene in typical periphyton and floc samples.

Compounds	WCA3 Periphyton (‰)	SRS2 Periphyton (‰)	SRS2 floc (‰)	WCA3 floc 1 (‰)^a^	WCA3 floc 2 (‰)^a^	WCA3 floc 3 (‰)^a^	WCA3 floc 4 (‰)^a^
C_27:3_	—	—	—	−36.8	—	—	—
C_27:2_	—	—	—	—	—	—	—
C_28:3_	—	—	—	−39.0	—	−35.5	—
C_28:2_	—	—	—	—	—	—	—
C_29:3_	−36.8	—	−37.6	−37.9	−38.0	−36.9	−37.5
C_29:2_	−38.3	−37.6	—	−38.0	−36.7	−35.6	—
C_30:3_	—	—	—	−37.5	−37.9	—	—
C_30:2_	—	−37.0	—	−37.1	−40.0	—	—
C_31:2_	—	—	—	−37.0	−39.9	—	—
C_32:2_	−37.4	—	—	−36.9	−38.2	—	—
Lycopadiene	−36.1	—	−35.5	—	—	—	−35.7

Note: ^a^denotes slough floc. “−” = not determined.

## Discussion

### Co-occurrence of *B. braunii* (A, B, L) indicated by *n*-alkadienes, *n*-alkatrienes, and lycopadiene in the Everglades

Lycopadiene has been reported as a specific biomarker for race L of *B. braunii*^4^, while botryococcenes have been suggested to derive from race B of *B. braunii* in the Everglades^18^. *n*-Alkadienes and *n*-alkatrienes were not detected in floc and surface soil at the mangrove-dominated estuarine locations^27^, nor in the leaves or roots of dominant plant species across the Everglades ecosystem^22,28^. *n*-Alkadienes and *n*-alkatrienes have been reported in insect wax lipids, but they usually cover higher carbon chain lengths up to C_39_^29^. Odd numbered carbon (poly) unsaturated *n*-alkenes in the range C_23_–C_31_ have been characterized in the chlorophyte *Chlorella emersonii*^30^, the diatom *Rhizosolenia setigera*^31^, and two marine eustigmatophytes^32^. C_29_, C_31_ and C_33_ alkenes with one to four double bonds are also produced by some haptophytes, such as *Emiliania huxleyi*, *Isochrysis galbana*, *Gephyrocapsa oceanica* and *Chrysotila lamellosa*^33–36^. However, the *n*-alkadienes and *n*-alkatrienes detected in this study are all from only the freshwater wetland locations, and thus should not be derived from haptophytes. No significant correlations (concentration based) were observed between *n*-alkadienes, *n*-alkatrienes, and the C_20_ HBI (highly branched isoprenoid) across the whole sample set (n = 98, *P* > 0.05), excluding cyanobacteria as the major source of *n*-alkadienes, *n*-alkatrienes detected^20,37^. Therefore, we suggest that the *n*-alkadienes and *n*-alkatrienes detected in this study likely derive from the A race of *B. braunii*^5^. Combining the botryococcenes and lycopadiene produced by the B and L races of *B. braunii*, the co-occurrence of three races (A, B and L) of *B. braunii* seems possible.

No significant correlations exist among the abundances of biomarkers of different races of *B. braunii*, which could be caused by: (1) variations in the populations of each race of *B. braunii* across our study area, (2) differences in the hydrocarbon concentration in each race, and (3) different physiological states for each race^4^. Similar results have also been observed in another study^1^. Mixtures of *cis n*-alkadiene and triene(s) or *cis*/*trans* dienes (without triene) covering the carbon chain range from C_25_ to C_31_ have been characterized in the A race of *B. braunii*^3,6,7,38,39^. *Cis*-dienes have been suggested to be produced via an elongation-decarboxylation mechanism with oleic acid as the direct precursor^40^. The L race of B. *braunii* can produce lycopadiene^5^. Recently, the microfossils of *B. braunii* have been observed in soil cores of tree islands, and floc at WCA3 area in the Everglades^16^, providing evidence for the existence of *B. braunii* in this ecosystem. Unfortunately, no specific race of *B. braunii* was described in previous studies.

Lower δ^13^C values were obtained for *n*-alkadienes, *n*-alkatrienes, and lycopadiene, which are similar to those observed previously for botryococenes^18^, suggesting that these compounds are likely produced by *B. braunii* utilizing at least partially recycled (^13^C depleted) CO_2_ from organic matter degradation as their carbon sources rather than atmospheric (^13^C enriched) CO_2_^21^. Similar lower δ^13^C values have also been observed for C_20_ HBIs (for cyanobacteria) in the freshwater Everglades periphyton and floc. Although the δ^13^C values of the *n*-alkadienes and *n*-alkatrienes, to our best knowledge, have not yet been reported, the δ^13^C values for the biomarkers of *B. braunii* are diverse^41^. The δ^13^C values of botryococcenes (or botryococcanes) and lycopadiene-derived compounds are reported from −37.4‰ to −10.6‰ and −29.0‰ to −21.0‰, respectively^9,18,41,42^. Even though, Boreham *et al*.^43^ stated that the large range of δ^13^C values may not be fully expressed due to differences of internal diffusion rates of CO_2_, this wide range of δ^13^C values is at present not clear^43^.

### Environmental controls of *B. braunii* biomarkers and their implications in the Everglades

Although *B. braunii* is known to be sensitive to environmental changes^42^, and botryococcenes have been suggested to be applied as a proxy for eutrophication, the lack of correlations between nutrients and *n*-alkadienes and *n*-alkatrienes suggest that they seem not to be indicators for eutrophic conditions in this freshwater wetland. Actually, *Botryococcus* was also not suggested to directly reflect nutrient status of waters in the Everglades^17^.

In contrast to nutrients, hydroperiod seems to be one of the controlling factors for the distribution of *n*-alkadienes and *n*-alkatrienes. Significant higher abundances of *n*-alkadienes and *n*-alkatrienes were observed in the ridge than slough floc, which could be explained by the following reasons: (1) the A race of *B. braunii* has the ability to float due to its high lipid concentrations^44^, which leads to its enrichment in the slough environment^17^; and (2) more diagenetic degradation of *n*-alkadienes and *n*-alkatrienes occurs in the ridge environment due to stronger oxidation. In this study, *n*-alkadienes and *n*-alkatrienes were only observed in the floc at locations SRS2 and WCA3, in agreement with higher concentrations of botryococcenes reported at these two sites^18^. This could be mainly attributed to longer hydroperiod (WCA3 and SRS2), and lower water flow velocities (WCA3), resulting in reduced floc transport (Table 1). The longer hydroperiod could induce sub-oxic or anoxic conditions in the floc layer, and thus decrease carbon mineralization rates. However, other factors may also contribute to the concentration difference among different sites, such as the composition of periphyton and these require further investigations (Table 1)^45^.

*n*-Alkadienes and *n*-alkatrienes were only detected in WCA3 slough soils/sediments (Fig. 3), but not in all SRS and TSPh sites, which could likely be due to: (1) a more complex microbial composition in periphyton and floc compared to soils^13,14^, (2) limited incorporation of periphyton and floc into soils, or (3) early diagenetic reworking or microbial degradation of these compounds by heterotrophs such as bacteria and fungi^46^. Several sulfur-containing compounds and two thiophenes both with 20 carbon atoms (3-methyl-2-(3′,7′,11′-trimethyldodecyl)thiophene and 3-(4′,8′,12′-trimethyltridecyl)thiophene were detected in most of the floc samples (data not shown), suggesting anoxic or sub-oxic conditions. If early diagenetic reduction of the unsaturations is one of the factors accounting for the absence of *n*-alkadienes and *n*-alkatrienes in all deeper soils/sediments, part of the C_27_–C_33_ *n*-alkanes detected in sediments of the Florida wetland could also be derived from the *n*-alkadienes and *n*-alkatirenes^47^. However, further investigation is needed. Lycopadiene was not detected in surface and deeper soils, likely due to: (1) a much lower amount of lycopadiene produced, or (2) diagenetic transformation of lycopadiene into higher molecular weight compounds^47^. However, lycopadiene was reported in a few studies including freshwater lake sediments from the Holocene^1^ and an oil shale from the Pliocene^47^. In addition, a monoaromatic lycopane derivative was reported from the Messel oil shale^9^, and kerogen fractions of samples from oil shale layer 4 in the Eocene Huadian Formation, NE China^48^. Adam *et al*.^9^ proposed that this compound could be a specific biomarker for race L of *B. braunii* in sediments deposited under freshwater and/or brackish conditions^9^. Though analyzing a Holocene freshwater lake sediment core, Zhang *et al*.^1^ suggested that *n*-alkadienes, botryococcenes and lycopadienes can survive in oxic sediments for several decades, and the down core variation in these lipids likely reflects changes in environmental conditions either favoring the bloom or near-extinction of *B. braunii*^1^. However, this present study shows that botryococcenes were widely detected, and *n*-alkadienes and *n*-alkatrienes were rarely present, while lycopadiene was not detected in the surface and deeper soils of this subtropical freshwater wetland. This possibly suggests either their general rapid diagenetic reworking, or more likely to a recent increasing contribution of Botryococcus-derived organic matter input in the Everglades. Further studies are needed to address the other factors controlling the distribution of these biomarkers in order to better use them as indicators of the *B. braunii* community structure in the Everglades.

## Conclusions

A series of long chain *n*-alkadienes and *n*-alkatrienes, botryococcenes and a lycopadiene were detected in periphyton and floc across the freshwater wetlands of the Florida Everglades, USA, suggesting the co-existence of all three races of the green alga *B. braunii* (A, B and L). Similar low δ^13^C values were observed for *n*-alkadienes and *n*-alkatrienes, lycopadiene and botryococcenes, suggesting that the source organisms (*B. braunii*) at least partially utilize recycled CO_2_ produced from respired organic matter rather than atmospheric CO_2_ as the carbon sources. The concentrations and molecular distributions of these compounds were shown to decrease from floc to periphyton. *n*-Alkadienes, *n*-alkatrienes and lycopadiene were not found in soils, suggesting a recent contribution of these compounds likely due to the blooming of *B. braunii*.

The abundance of these compounds does not correlate with both bulk N and P concentrations in floc samples or surface water, suggesting that nutrients may not be the controlling factors for the distributions of these compounds in this ecosystem. In contrast, slough floc contains significantly higher amounts of *n*-alkadienes and *n*-alkatrienes than ridge floc. Thus, hydroperiod could be one of the controlling factors for the abundances of *n*-alkadienes and *n*-alkatrienes within this freshwater wetland. However, further investigation is needed to refine the application of these biomarkers asl indicators of community structure of *B. braunii* in the Everglades.
